# Delivering collaborative mental health care within supportive housing: implementation evaluation of a community-hospital partnership

**DOI:** 10.1186/s12888-021-03668-3

**Published:** 2022-01-13

**Authors:** Lucy C. Barker, Janet Lee-Evoy, Aysha Butt, Sheila Wijayasinghe, Danielle Nakouz, Tammy Hutcheson, Kaela McCarney, Roopinder Kaloty, Simone N. Vigod

**Affiliations:** 1 Women’s College Hospital, 76 Grenville Street, 7th Floor, Toronto, ON M5S 1B2 Canada; 2 Department of Psychiatry, University of Toronto, Toronto, Canada; 3 Institute for Health Policy, Management and Evaluation, University of Toronto, Toronto, Canada; 4 Unity Health, Toronto, Canada; 5 Department of Community and Family Medicine, University of Toronto, Toronto, Canada; 6 YWCA-Toronto, Toronto, Canada; 7 The Jean Tweed Centre, Toronto, Canada

**Keywords:** Supportive housing, Collaborative, Psychiatry, Community-hospital partnership

## Abstract

**Background:**

Approaches to address unmet mental health care needs in supportive housing settings are needed. Collaborative approaches to delivering psychiatric care have robust evidence in multiple settings, however such approaches have not been adequately studied in housing settings. This study evaluates the implementation of a shifted outpatient collaborative care initiative in which a psychiatrist was added to existing housing, community mental health, and primary care supports in a women-centered supportive housing complex in Toronto, Canada.

**Methods:**

The initiative was designed and implemented by stakeholders from an academic hospital and from community housing and mental health agencies. Program activities comprised multidisciplinary support for tenants (e.g. multidisciplinary care teams, case conferences), tenant engagement (psychoeducation sessions), and staff capacity-building (e.g. formal trainings, informal ad hoc questions). This mixed methods implementation evaluation sought to understand (1) program activity delivery including satisfaction with these activities, (2) consistency with team-based tenant-centered care and with pre-specified shared lenses (trauma-informed, culturally safe, harm reduction), and (3) facilitators and barriers to implementation over a one-year period. Quantitative data included reporting of program activity delivery (weekly and monthly), staff surveys, and tenant surveys (post-group surveys following tenant psychoeducation groups and an all-tenant survey). Qualitative data included focus groups with staff and stakeholders, program documents, and free-text survey responses.

**Results:**

All three program activity domains (multidisciplinary supports, tenant engagement, staff capacity-building) were successfully implemented. Main program activities were multidisciplinary case conferences, direct psychiatric consultation, tenant psychoeducation sessions, formal staff training, and informal staff support. Psychoeducation for tenants and informal/formal staff support were particularly valued. Most activities were team-based. Of the shared lenses, trauma-informed care was the most consistently implemented. Facilitators to implementation were shared lenses, psychiatrist characteristics, shared time/space, balance between structure and flexibility, building trust, logistical support, and the embedded evaluation. Barriers were that the initial model was driven by leadership, confusion in initial processes, different workflows across organizations, and staff turnover; where possible, iterative changes were implemented to address barriers.

**Conclusions:**

This evaluation highlights the process of successfully implementing a shifted outpatient collaborative mental health care initiative in supportive housing. Further work is warranted to evaluate whether collaborative care adaptations in supportive housing settings lead to improvements in tenant- and program-level outcomes.

**Supplementary Information:**

The online version contains supplementary material available at 10.1186/s12888-021-03668-3.

## Background

Stable housing is important for achieving and maintaining long-term health and stability for individuals with a variety of mental illnesses. Barriers to stable housing include unaffordability, stigma from potential landlords, and a lack of mental health supports in housing settings [1, 2]. There is strong evidence that permanent supportive housing, defined as “safe and stable housing environments with voluntary and flexible supports and services to help people manage serious, chronic issues such as mental and substance use disorders” [3] can increase housing stability [4]. It is also associated with improved psychiatric outcomes, such as reduced drug and alcohol misuse, and reduced hospitalization risk, particularly in women [3, 5].

Voluntary mental health supports are a key component of supportive housing, however, there is evidence of unmet need in mental health care delivery across settings [6]. In a U.S. study of permanent supportive housing agencies (*n* = 23), less than two-thirds offered on-site mental health services; these were mostly provided by outside agencies, with reports of poor communication between agencies and frontline staff [6]. In a Canadian survey of 96 housing and 186 community-based mental health service workers, 53.5% reported that integrated mental health and housing services was an area of unmet need [1]. New approaches to mental health service provision in permanent supportive housing are needed. Multidisciplinary team-based approaches with strong supervision, robust staff supports, and ongoing staff training are suggested approaches to providing mental health services in supportive housing settings [7].

Collaborative care is a team-based multidisciplinary approach to mental illness management. The approach involves structured care plans, scheduled follow-ups, and systematic inter-professional communication [8, 9]. Key elements include patient self-management support (e.g. psychoeducation), clinical information systems use (to facilitate information flow between team members), delivery system redesign (i.e. redefinition of work roles to support collaborative care), provider decision support (i.e. integrated specialist input), linkages with community resources, and organizational support at a leadership level [10]. Collaborative care models – originally designed for treating mental disorders such as depression and anxiety in primary care [9] – have been successfully adapted in other populations, including for alcohol and opiate use disorders [11], and individuals experiencing socioeconomic disadvantage [12]. In shelters, a shifted outpatient collaborative care model with collaboration occurring directly between psychiatrists and shelter staff (without integrated primary care) appears as effective as traditional models where psychiatric care is integrated into on-site primary care infrastructure [13]. Collaborative care is an attractive model for permanent supportive housing settings due to existing infrastructure (e.g. housing support teams) and the opportunity for individualization of supports and services to support complex populations. Shifted adaptations hold promise for housing settings where levels of on-site primary care may vary. Despite this, we found that applications of collaborative approaches in permanent supportive housing have rarely been reported.

In the current project, we developed and preliminarily evaluated a shifted outpatient collaborative mental health care adaptation in a permanent supportive housing setting in a large urban centre in Canada. The intervention was developed and delivered by stakeholders (managers, physicians, and nurse practitioners) from a housing agency, a community mental health agency, and an academic hospital. By agreement of stakeholders, the intent was for development and implementation of the intervention to be based on principles of team-based and tenant-centred care, underpinned by trauma-informed [14, 15], culturally safe [16], and harm-reduction lenses [17]. In an iterative process evaluation, we aimed to understand and improve implementation over the first year of the initiative, specifically to answer the following questions (1) What activities were delivered, and what were staff/tenant/stakeholder perspectives of these activities? (2) Were these activities consistent with the principles of team-based tenant-centered care and with the shared lenses? (3) What were the barriers and facilitators to implementing the intervention? Given the large existing body of literature related to supportive housing [3], and scant literature on the potential role for collaborative psychiatric care integrated into supportive housing settings, the focus of this evaluation was specifically on the addition of the collaborative psychiatric care.

## Methods

### Design

A mixed-methods process evaluation was used to evaluate the implementation of the initiative over its first year (May 2019 to May 2020). The evaluation was designed in line with the Medical Research Council guidance on process evaluation for complex interventions [18], and reported according to the Standards for Reporting Implementation Studies (StaRI) Statement [19]. Evaluation questions were formulated to address fundamental process evaluation metrics (according to Saunders et al. [20]): Aim 1 addressed dose delivered and dose received (including satisfaction), Aim 2 addressed fidelity, and Aim 3 addressed context (reach and recruitment were not formally assessed) (Fig. S1). As the focus was on the process of implementation, no effectiveness measures were planned. When complex interventions are implemented in new contexts, it is expected that some elements of the intervention will need to be tailored over time [18]. The evaluation was therefore intended to be flexible and to capture and facilitate any changes [18]. The evaluation design and all iterative changes were discussed, reviewed with, and approved by the stakeholders as the implementation progressed.

### Context

The intervention was developed in partnership between the YWCA Elm Centre and Women’s College Hospital (WCH). The YWCA Elm Centre is a not-for-profit housing complex with 300 mixed housing units for women and gender diverse people and their children (85 supportive housing units for individuals with complex mental health needs, 50 Indigenous-specific units, and 165 affordable rental units). Existing Elm Centre services were provided by (1) the YWCA, (2) the Jean Tweed Centre, and (3) WCH. The YWCA provides housing supports (eviction prevention), community engagement, and individualized mental health and substance use supports as needed for tenants of all 300 units. The Jean Tweed Centre, a community based substance use and mental health agency, provides case management and some nurse practitioner-led primary care to tenants of the supportive housing and Indigenous-specific units. WCH, a nearby academic hospital, had formed a primary care partnership with the Elm Centre in 2015 to support tenants of supportive housing and Indigenous-specific units (50). Initially, the WCH family physician provided on-site primary care, however by 2018 she had successfully connected most tenants with off-site primary care providers, so the role had transitioned to one of system navigation, liaison, coordination, and advocacy. Some tenants had access to off-site psychiatric services (e.g. assertive community treatment), but most tenants did not have ongoing psychiatric care. An on-site psychiatrist from WCH previously provided direct consultation and follow-up onsite at the Elm Centre, but uptake and integration were poor, and there was no formal collaboration between the psychiatrist and housing, case management, and primary care providers. In 2018, WCH and YWCA-Elm decided to pursue a new model and WCH committed to 3 years of funding for psychiatric indirect care (CA$25,000 per year for a half-day per week of indirect care), program development (e.g. purchase of reference materials for use with staff and tenants), and evaluation.

### Targeted sites and participants

A stakeholder group consisting of managers, nurse practitioners, and physicians from YWCA-Elm, the Jean Tweed Centre, and WCH participated in designing and implementing the initiative. Stakeholders came from diverse professional and personal backgrounds. YWCA-Elm and Jean Tweed Centre staff who worked with tenants (e.g. case managers, community engagement workers) participated in the intervention. Tenants in supportive housing and Indigenous-specific units were the focus of the intervention, however some aspects of the initiative (e.g. psychoeducation sessions) were open to all tenants.

### Intervention description

**Fig. 1:**
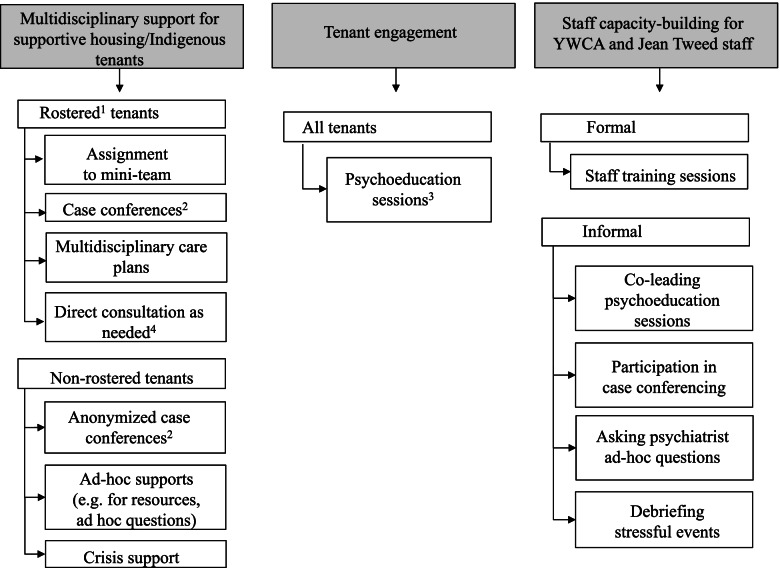
Initiative design. ^1^Rostered tenants were tenants in supportive housing/Indigenous-specific units who had complex mental health needs, required additional support, and who consented to information sharing between WCH, the YWCA, and the Jean Tweed Centre. ^2^Case conferences involved the psychiatrist, YWCA staff, Jean Tweed Centre staff, and, when available, primary care (WCH family physician/Jean Tweed nurse practitioners). ^3^Psychoeducation sessions served to introduce the psychiatrist, improve mental health knowledge, and destigmatize mental health. They were facilitated by YWCA staff, with the psychiatrist (and the family physician, when available) providing expertise on the chosen topic. ^4^Direct consultation was either 1:1 with the tenant and psychiatrist, or when requested by the patient and team, was joint with the tenant, psychiatrist, and staff from YWCA and/or Jean Tweed

The intervention was designed by stakeholders from the three partner organizations, and desired outcomes included meeting tenants’ mental health care needs, enhanced safety, trauma-informed and culturally-safe care, better sense of agency and support among staff, and sustainability in care (see logic model, Fig. S2). Since most individuals had external primary care providers, a modified shifted collaborative care model [13] was envisioned in which the psychiatrist would collaborate directly with Elm Centre housing/case management staff in addition to on-site primary care supports. The plan was to iteratively adapt the intervention during the course of the project. The program activities fell into three categories (1) multidisciplinary support for tenants, (2) tenant engagement, and (3) building staff capacity (Fig. 1). Within (1) multidisciplinary support, a rostering system was used to identify tenants who (a) lived in supportive housing or Indigenous-specific units, (b) required enhanced mental health supports, and (c) consented to having YWCA, the Jean Tweed Centre, and WCH collaborate around their care. Rostered tenants were those who met all three criteria. Of note, acceptance of medication was not a condition to being rostered. Initially, the plan was for rostered tenants to be assigned a “mini-team”, consisting of a YWCA community engagement worker and a Jean Tweed Centre case manager. The psychiatrist supported rostered tenants via a mixture of indirect and direct care (Fig. 1) and supporting external referrals to intensive services when necessary (e.g. assertive community treatment [21]). Psychiatric care included recommendations for medications and psychotherapy, crisis supports, as well as advocacy interventions (e.g. supporting refugee processes). Depending on the circumstance, within direct care tenants could meet one-on-one with the psychiatrist, or have a joint meeting including YWCA and/or Jean Tweed staff. The team recognized that some tenants would not need formal rostering but would still benefit from support, or would decline formal rostering due to a preference to keep housing supports and mental health supports separate, stigma around mental illness, or previous adverse experiences with mental health providers. Staff could still solicit support for these “non-rostered” tenants (e.g. ask deidentified questions, ad hoc supports). Within (2) tenant engagement, the main planned activity was group-based psychoeducation open to all tenants in the building. The WCH family physician had previously successfully engaged tenants in group-based health education so this format was used for psychoeducation. Within (3) staff capacity-building, training sessions for staff were led by the psychiatrist alone or co-led with the WCH family physician, with the plan for topics to be chosen collaboratively with staff. Informal staff capacity building was also anticipated through participating in multidisciplinary tenant support and tenant engagement.

It was a priority that the initiative be flexible and attuned to reflect the local context, including the large proportion of Indigenous tenants [22]. Both Indigenous and non-Indigenous tenants had experienced high rates of trauma, discrimination, and substance use. Trauma-informed [14, 15], culturally safe [16], and harm-reduction lenses [17] were expected to enhance the patient-centeredness of the program, and the shared lenses were expected to facilitate collaboration amongst team members. Trauma-informed care was operationalized through having a psychiatrist specialized in trauma, trauma-informed care plans, and education about trauma integrated into both psychoeducation for tenants and training sessions for Elm Centre staff [14, 15]. Culturally safe care was operationalized by reciprocal education and knowledge-sharing with staff (including Indigenous staff), considering cultural factors within care plans, maintaining an awareness of sociopolitical factors (e.g. history of colonization), and considering referral to culturally-specific services as appropriate [16]. Principles of harm reduction were incorporated by meeting clients where they were in their recovery journey, and considering concurrent management of addictions and psychiatric illness within care plans in line with tenant goals (including referral to outside harm reduction-informed services as needed) [17].

### Implementation strategies

The implementation strategies were multi-pronged, and were identified in accordance with the Expert Recommendations for Implementing Change (ERIC) [23, 24]. We formed an academic partnership, identified and prepared stakeholder champions from all three organizations, and held regular implementation team meetings with identified stakeholders. New funding was accessed for intervention components not covered by existing streams (e.g. activities not billable under provincial health insurance). The intervention and evaluation were designed to be adaptable and tailored to the housing context, and the services were delivered on-site. We used iterative evaluation strategies, specifically purposefully re-examining the implementation (monitoring progress and adjusting practices to improve the intervention), and identifying barriers and facilitators. We conducted educational meetings with the staff prior to implementation, initially created new teams (mini-teams), and created a collaborative learning environment to facilitate implementation among staff. Although we were not able to change the record systems to allow for shared documentation due to privacy/consent and individual organization requirements, we did streamline processes for tenants to provide consent for information-sharing across organizations.

### Evaluation data sources

Data sources were designed to be pragmatic, and, whenever possible, use data already collected by the program. Data sources were (a) program documents consisting of stakeholder meeting minutes (~monthly), weekly psychiatrist documentation (detailed de-identified report on all activities, e.g. number of case conferences) and a de-identified care plan log for rostered tenants (components of care plans including medication management, psychosocial support), (b) staff surveys distributed at 3 and 9 months, (c) focus groups with staff and stakeholders at 6 and 12 months (semi-structured), (d) an all-tenant survey delivered at 12 months, and (e) tenant surveys following psychoeducation sessions. Interviews with tenants were planned, but not completed due to a lack of uptake from tenants. Surveys for external primary care providers were initially planned, but were deferred as the initial focus was on internal collaborations.

### Outcomes

For Aim 1, the delivery of activities was assessed quantitatively using weekly psychiatrist documentation (number of each program activity delivered each week) and stakeholder meeting minutes (planned changes to activities) (Fig. S1). Staff, tenant, and stakeholder perspectives of activities were assessed quantitatively using staff surveys (Likert 1-5 satisfaction rating) and tenant post-psychoeducation session surveys (Likert ratings of questions related to group utility, feeling respected, and satisfaction with groups, Likert 1-5) and qualitatively using staff and stakeholder focus groups (questions on experiences and opinions of activities) and open-ended feedback on the all-tenant survey. For Aim 2, to assess consistency with team-based patient-centered care, we (a) documented the frequency of team-based activities (e.g. case conferences, capacity-building, co-led tenant groups) and non-team-based activities (e.g. direct consultation with tenants) using weekly psychiatrist documentation, (b) documented the providers involved in care plans using the log of rostered tenants, (c) solicited quantitative ratings of collaboration, engagement, tenant-centeredness, and consistency with each of the shared lenses (trauma-informed, culturally safe, harm reduction) on staff surveys (each Likert scale 1-5), (d) asked questions related to collaboration, tenant-centeredness, and shared lenses during staff/stakeholder focus groups (qualitative), and (e) solicited tenant perspective using open-ended questions on the all-tenant survey. For Aim 3, we used qualitative data from stakeholder meeting minutes, staff/stakeholder focus group data, and free-text tenant survey responses to assess barriers and facilitators to implementation. To capture additional contextual information and information about any changes to the initiative, we used qualitative data from stakeholder meeting minutes (main data source) as well as information from psychiatrist documentation and staff/stakeholder focus group data. Outcomes of the intervention (e.g. change in tenant mental health) and economic evaluation were outside the scope of the study.

### Sample size and recruitment

The evaluation was designed to ensure that tenants, staff, and stakeholders felt comfortable sharing information with the evaluation team (e.g. managers not having access to staff feedback, ensuring that tenants knew the evaluation was not linked to housing status). Staff were invited to participate in surveys via email; the goal was for all staff (up to 14 individuals) to participate. Stakeholders and staff were invited to participate in focus groups via email; the aim was to conduct one stakeholder and one to two staff focus groups with 4-10 participants per group at each time point. Rostered tenants were to be invited to participate in interviews by their case managers/community engagement workers. Following psychoeducation groups, post-group surveys were distributed on paper to all interested participants. An all-tenant survey was distributed on paper to 135 supportive housing and Indigenous-specific units under their door (or directly by staff in certain circumstances). Draws for gift card prizes (CA$25) were done for each of the staff surveys and the all-tenant survey.

### Analysis

Analysis was done throughout the evaluation to iteratively refine the implementation process. Quantitative data were analyzed using descriptive statistics in Excel. Likert-type responses were described using medians and interquartile ranges (IQR). Qualitative data were analyzed using thematic analysis, using the framework explication by Braun and Clarke [25]: (i) documents/transcripts were read and re-read for a broad understanding, (ii) documents/transcripts were examined closely and initial codes inserted, (iii) codes were grouped into potential themes, (iv) themes were reviewed and mapped conceptually, and (v) refined and grouped into themes/sub-themes. The aim of the analysis was to deductively identify themes related to participants’ qualitative experiences of the program and provide further contextual information. Documents and transcripts were coded by two team members (LB/AB), and discrepancies were discussed to reach consensus. Qualitative data was analyzed using NVIVO software. The two methods were then triangulated; quantitative and qualitative data were interpreted together to maximize the information and perspectives available for each study aim. No subgroup analyses were planned.

### Ethical considerations

Ethics approval was obtained through Women’s College Hospital Ethics Assessment Process for Quality Improvement Projects (WCH APQIP). All methods were conducted in accordance with relevant guidelines and regulations.

## Results

Over the course of the year, there were a total of 9 YWCA staff (4-6 at one time, with turnover) and 8 staff from the Jean Tweed Centre (4-6 at one time); all interacted with the psychiatrist through case conferences, staff training, and ad hoc support. Thirteen tenants were formally rostered. As expected, the rostering process posed barriers for those tenants who were ambivalent about receiving integrated support, and the total number of tenants who received support from the initiative in other ways (e.g. de-identified case conference via their case manager, crisis support) was not captured.

Staff participation was low in the 3-month survey (*n* = 3) but improved for the 9-month survey (*n* = 10). Three stakeholders and 11 staff participated in the 6-month focus groups, and four stakeholders and seven staff participated in the final (12-month) focus groups. Post-group surveys were distributed after two different psychoeducation groups and completed by seven and six tenants respectively. Only five tenants completed the all-tenant survey (March 2020, at the start of the COVID-19 pandemic).

### Aim 1 (program activities: delivery and satisfaction)

The psychiatrist was on site a median of 3 days per month (IQR 3-3.5). Each month, there were a median of 7 case conferences (IQR 4.5-7.5) and 2 psychiatric consultations (IQR 1-2) (Table 1). Psychoeducation groups for tenants and staff training sessions occurred approximately monthly. On staff surveys, satisfaction was neutral (median 3, IQR 3-4) (Table S1).

**Table 1:** Program activities August 2019-February 2020^a^

Activity	Median number per month (IQR)
Days on-site per month	3 (3-3.5)
Case conferences	7 (4.5-7.5)
Direct consultations with tenants	2 (1-2)
Psychoeducation sessions for tenants^b^	1 (0.25-1)
Teaching sessions for staff^c^	1 (1-1)

^a^Documentation started in August 2019 once ethics approvals were in place, and ended in February as March-April were during the COVID-19 pandemic

^b^Topics: Meet and greet, Mental wellness, Seasonal Affective Disorder

^c^Topics: Boundaries, risk assessment, case conferencing, personality disorders 1 &2, psychopharmacology, substance use disorders

Of the program activities, staff particularly valued the support of having an on-site psychiatrist and capacity-building through formal sessions and ad hoc questions: “… I’ve found that the knowledge sharing has been really useful with what she’s brought forward in terms of contemporary or current best practices” (Staff final focus group).

“I personally have been using Dr. X a lot for my own questions, and I have found that really, really helpful because there are just things that we just don’t know. As much as you Google something, she has more knowledge, a whole lot more knowledge, than Google can tell us” (Staff 6-month focus group).

Within support of tenants, advocacy for tenants was highlighted by staff as being particularly useful: “We have a number of tenants who are really unwell, and so being able to have Dr. X on hand to give us some feedback and more insight into what’s going on, it has just really helped us advocate in ways that have been very supportive for the tenants that we’re working with” (Staff final focus group).

Tenants who participated in the psychoeducation sessions reported they learned new information, that they were helpful to their lives and felt respected in the group, appreciated having it offered onsite and were likely or highly likely to attend another (median responses to all questions were 4-5) (Table 2).

**Table 2:** Tenant psychoeducational sessions

Topic	Mental wellness	Seasonal affective disorder
**Number of survey participants**	*N* = 7	*N* = 6
**Questions, Median (Interquartile range)**		
I learned something new in the group today^a^	4 (3-4)	4.5 (4-5)
The information and/or skills that were discussed in the group today will be helpful in my life^a^	4 (3.5-4)	5 (5-5)
I felt respected by other participants in the group^a^	4 (4-4.5)	4.5 (4-5)
I felt respected by the group facilitator(s)^a^	4 (4-5)	5 (4.25-5)
I appreciated having this group offered on-site at the Elm Centre (instead of somewhere else such as a hospital or health care clinic)^a^	4 (4-5)	5 (5-5)
I am glad I came to the group today^a^	4 (4-4.5)	5 (5-5)
How likely would you be to come to another group session?^b^	4 (4-5)	5 (5-5)

^a^Responses: 1 = Strongly disagree; 2 = Disagree; 3 = Neutral; 4 = Agree; 5 = Strongly Agree

^b^Responses: 1 = Very unlikely; 2 = Unlikely; 3 = Neutral; 4 = Likely; 5 = Very likely

### Aim 2 (consistency with team-based tenant-centered care and the shared lenses)

Consistent with the focus on team-based care, collaborative activities (case conferences, co-led tenant psychoeducation sessions, staff trainings) were more common than direct psychiatric consultations (Table 1). All rostered tenants (*n* = 13) had interdisciplinary care plans that involved medication management by their primary care provider/external psychiatrist and/or psychosocial care managed by a case manager/community engagement worker at the Elm Centre. Among rostered tenants, the number of case conferences ranged from 1 to 8 (median 3, IQR 1-3) and the number of psychiatric consultations/direct care sessions ranged from 1 to 4 (median 1, IQR 1-2) between August 2019 and February 2020. In staff surveys, the median ratings for collaboration, engagement, and patient-centeredness were generally positive (collaboration: median 3.5, IQR 3-4; engagement: median 4.5, IQR 4-5 at 9 months; patient centeredness: median 4, IQR 4-4 at 9 months) (Table S1).

In the focus groups, staff and stakeholders suggested that collaboration took time: “I feel like it’s built over time… It was a little rocky at the start in just trying to figure out what the mini teams are, what are the other roles [the psychiatrist]‘s going to be taking on here, whether that’s the capacity building pieces or the group work. I think as we start to move through some of those pieces and they become more consistent, it does feel more collaborative” (Staff 6-month focus group).

Tenant-centeredness was more complex. Initially, the initiative was felt to focus more directly on the needs of staff as opposed to on the needs of tenants: “I understand obviously the whole purpose for doing this is to support the community that we serve, even if I think that it has started off being more indirect in that they support us to support them” (Staff 6-month focus group).

Feedback on the all-tenant survey in month 10 suggested that integrated on-site services were in line with the needs of the tenants completing the survey: “I appreciate the way you have put together mental well-being services, since it has made grand improvement towards my healing, especially the resources have been useful as well as helpful. Also having help within the complex. Help coming in here from the outside is wonderful and touching” (Tenant, in survey response).

Of the three shared lenses, trauma-informed care was most highly rated on staff surveys (Table S1) and was the most universally and enthusiastically acknowledged as having been successfully implemented in qualitative feedback (Table S2). Cultural safety was a more complex lens to implement, as staff pointed out that psychiatry, which is embedded within a Westernized medicine paradigm, may be fundamentally incompatible with the worldviews of tenants. That said, staff felt that a culture of “respect” was consistently present which was important to cultural safety. Overall, staff also felt that the initiative successfully embodied a harm reduction lens.

### Aim 3 (facilitators and barriers to implementation)

Facilitators to implementation included (1) the pre-specified shared lenses, (2) the personal characteristics of the psychiatrist, including that she was seen as embodying the values of the Elm Centre, (3) shared time and space (joint team meetings, the psychiatrist using a shared office space with staff, and availability for real-time support when needs arose with tenants), (4) a balance between structure and flexibility in delivery of the initiative, (5) building trust over time, (6) logistical support from the organizations including funding, and (7) having an embedded evaluation to provide feedback and identify areas for change (Table 3).

**Table 3:** Facilitators and barriers to implementation

Facilitators	Barriers
**1) Shared lenses** “I think if someone who had come in and didn’t share those things we wouldn’t utilize them so I think that would make me feel comfortable with Dr. X, and so, therefore, the tenants feel comfortable, which is great.” (Staff 6-month focus group) **2) Personal characteristics of the psychiatrist** “I appreciate her way that she works. I don’t know if it would be the same with a different psychiatrist.” (Staff final focus group) **3) Shared time and space** **a) Joint team meetings** “I think having these joint team meetings has helped me in a way that I could feel like we’re all going to end up on the same page when dealing with certain clients. I think having never worked in this kind of situation before, in a partnership, it’s really been helpful to get everyone’s point of view and opinions and strategies.” (Staff final focus group) **b) Shared space** “She has also started sitting in our office with us, which I have found really great, and I know she actually said the same thing, that it has been nice because we’re very talkative in our office. That’s where we just bring things up, and having her there, she’s right there.” (Staff 6-month focus group) “Now, with her there, I’ll just be reading an email and be like, hey, can you answer this question? It’s just so much more accessible.” (Staff 6-month focus group) **c) Real-time support** “And I really appreciate the consultation supports that she can offer in real time. I find with community staff and community psychiatrists, there’s always such a lag or a period of time we have to wait to hear back. So, I really appreciate how connected and so in the loop she is and cares to be.” (Staff final focus group) **4) Balance between structure and flexibility** “I feel she’s been open too because I feel we’re almost all figuring it out together because I think part of it was she didn’t know exactly what it looked like here and exactly what it was going to be like. So, we have come to her with things, and she’s been like, sure, yeah and vice versa maybe so that has facilitated the project.” (Staff 6-month focus group) **5) Allowing time to build trust** “I can see it also from more and more staff willing to share more and more, even when there is an insult along the way because they needed to trust how you, as a psychiatrist, would be approaching the work. I don’t know, but to me that’s the most resounding success from creating community partnership.” (Stakeholder final focus group) **6) Logistical support from organizations** **7) Embedded evaluation** “I appreciate that you guys are doing focus groups and questionnaires that are all anonymous so that we can actually have open and honest conversations.” (Staff 6-month focus group) “There is something about structuring reflection and qualitative and quantitative wrap-up and stuff that I think is very, very crucial for us to have this dialogue.” (Stakeholder final focus group)	**1) Initial model was driven from leadership via from ground up** “The setup of it has made me feel a little bit like the tenants don’t have a say in what this looks like at all because it really did come from the top down.” (Staff 6-month focus group) “I think when this collaboration started, I think a lot of the building up of what it was intending to be was done between management, and then some of the higher ups in the various organisations. I remember when it was starting, as a frontline worker, not having a lot of information or clarity about what the program would be, what it would look like, and not necessarily being asked for frontline feedback either.” (Staff final focus group) **2) Confusion and mismatch in initial referral process a) Initial confusion re referral processes** “But I think the process as a whole has been very confusing, specifically around, I think, how and when we’re supposed to access her, because there have been multiple things suggested on how we’re able to use her as a resource here.” (Staff 6-month focus group) b**) Mismatch between tenants who want to engage and who stakeholders want to engage**“…but it’s like, whoever is yelling the loudest during that time gets the spot. And I felt like that’s how those people were chosen. Not necessarily because we felt that it was a service that they would engage in or exploring other options.” (Staff 6-month focus group) **3) Different workflows across organizations**“With the mini-team, I think the structural challenges were mimicking the general bigger program so privacy, confidentiality, different roles that seemingly have a lot of inherent tensions, even when ideally they shouldn’t, but very understandably do in how we sit, and how we form to create a constellation of supports.” (Stakeholder final focus group) **4) Staff turnover** “… there was a lot of turnaround with staff from the workers’ perspective, from management’s perspective and every time there is a new player the dynamic shifts. Not that we have to start from scratch but there was almost taking it back to the basics, which I think we’ve done and done well but we’re still trying to build that up and maintain the expectations and learning from that, what’s working and what’s not working.” (Stakeholder 6-month focus group)

Barriers to implementation identified in the first 6 months were subsequently addressed. In response to concerns from staff that the model had been developed by leadership without adequate input from front-line staff and tenants, more efforts were made to engage tenants (via an all-tenant survey, and post-group surveys) and to respond to staff feedback. Initially, staff reported confusion about referral processes, and felt that the process for choosing patients to be rostered was not appropriate. As a result, referral processes were clarified and opened up to allow for more opportunities for staff to connect tenants to the initiative. Another barrier was different workflows across organizations which was a barrier to implementing mini-teams; as a result, the mini-team idea was discarded in favour of a more flexible team-based approach. There was turnover in staff and stakeholders over the course of the year. Consent to communicate was initially anticipated to be a major barrier, however, this did not bear out in the evaluation; when tenants did not consent for information sharing, staff used the psychiatrist for support in other ways, for example by asking general questions or through de-identified case consultation. In the all-tenant survey, the respondents were generally enthusiastic about service providers from different organizations working together “I welcome all the support I can receive to enhance my healing and well-being” (Tenant, in survey response).

### Fidelity to the intervention components and implementation strategy

All three main areas of the intervention (multidisciplinary support for tenants, tenant engagement, and building staff capacity) were implemented through the year. As described above, within multidisciplinary support for tenants, the main change was replacing pre-specified “mini-teams” with a more flexible team-based approach. Psychoeducation sessions and staff training were implemented as planned.

With respect to the implementation strategy, stakeholder engagement, funding, supporting clinicians/staff, engaging tenants, evaluative and iterative strategies were implemented as planned. Along with the change in mini-teams, we added a referrals pathway document for staff to facilitate implementation of the new approach. Another change infrastructure element during the year was moving the day of the existing YWCA/Jean Tweed Centre joint staff team meeting so that the psychiatrist could consistently attend to allow for large group case conferences.

### Contextual findings

Having the collaborative care delivered on-site in the housing setting was key to the intervention, and bought up the unique “vulnerability of working in someone’s home” (Stakeholder 6-month focus group). Being on-site was seen as a facilitator to connecting tenants to care when they are at a moment for readiness to connect: “And I think one nice thing about being here and in housing is that we’re more able to catch people at times that they might be more amenable to speaking” (Staff 6-month focus group). Having the psychiatrist on-site facilitated collaboration with YWCA and Jean Tweed Centre staff, and she became more embedded in day-to-day operations; for example, she organically started working part time out of the case management office which enhanced collaboration.

### Contextual changes impacting implementation

Due to the COVID19 pandemic, regular in-person processes were disrupted in mid-March 2020. The psychiatrist continued to provide ad-hoc virtual support through the initial transition. The final focus groups were delayed and were virtual as a result of the pandemic (June 2020). The COVID-19 pandemic impacted the all-tenant survey as Ontario’s State of Emergency was announced during the collection period (March 13-27, 2020). Another contextual change was related to staff and stakeholder turnover. This may have affected initiative elements including team-based care (e.g. changing team composition) and staff capacity building (e.g. difficulty scaffolding learning). Due to unexpected circumstances, there were temporary changes to primary care involvement (including a period with no family physician); this limited the ability to fully explore the possibilities of collaboration with on-site and external primary care within the first year.

### Harms/unintended effects

Although no harms were identified, several potential issues were raised in the focus groups. While some staff spoke about providing psychiatric care on-site as “creat[ing] more of an opportunity for trust” (Staff 6-month focus group), others raised concerns about stigma related to having an on-site psychiatrist (“…I think in some ways it does contribute to that stigma around why do we need a psychiatrist here, are there crazy people in the building, what’s going to happen, kind of thing.” (Staff final focus group)). Another factor that arose in providing mental health care on-site was balancing roles of landlord (the YWCA) with supports provided by all three agencies. As one stakeholder put it “…how to reconcile landlord and supports on site when there is an inherent tension in that: relationship by virtue of eviction prevention work that needs to be done.” (Stakeholder 6-month focus group). In the tenant survey, there were favourable responses to having services on-site, however very few tenants returned the survey, and we cannot rule out harms or unintended consequences.

## Discussion

In this evaluation, we found that, with some responsive changes, we were able to successfully implement a modified shifted outpatient mental health collaborative care initiative within a women-centered supportive housing setting. Collaboration built over time and, consistent with the vision of the intervention, collaborative activities (e.g. case conferences) were more frequent than direct patient care. Of the program elements, capacity-building for staff and psychoeducation sessions for tenants were highlighted as particularly helpful. We identified several elements that enabled the implementation, including having shared pre-specified lenses that were relevant to the tenant population, a psychiatrist well-suited to the context, creating opportunities for shared time and space, balancing structure and flexibility, allowing time to build trust, and having logistical organizational support as well as an embedded evaluation. Several barriers arose during implementation, many of which were addressed through responsive changes.

The need for mental health care is high within supportive housing settings, and is not always addressed by existing services [1]. While multidisciplinary team-based approaches have been proposed [7], they have rarely been described, and examples that specifically include support from a psychiatrist are sparse in the published literature [26, 27]. In this evaluation, we described the successful integration of a psychiatrist within a women-centered supportive housing complex, and in particular were able to capture some of the adaptations needed to provide responsive team-based care in this setting. While the initiative was collaborative, the end product is a significant departure from a traditional collaborative care model [28]. Our initiative reflects many elements of a shifted outpatient collaborative care model previously described in a shelter setting in which the psychiatrist engaged in indirect patient discussion and educational support with shelter staff [13]. The biggest change in the initiative during implementation was from a structured “mini-team” approach for rostered tenants, to a flexible team-based approach that allowed for more informal supports. Although this flexibility strays further from traditional collaborative care models [28], it is in line with the principles of supportive housing where flexibility and tenant choice is paramount when delivering services [3]. It was hoped that the initiative could support the tenants with the most complex mental health needs; sometimes, however, these tenants did not consent to be seen by the psychiatrist, and rostering was voluntary. Because the psychiatrist was not on-site every day (or night), the ability of the psychiatrist to respond to acute crises was limited. Instead, support for these tenants was often indirect, e.g. through providing staff with resources and information, as well as advocating for more comprehensive mental health supports in the community (e.g. assertive community treatment).

While the focus of this implementation evaluation was on the unique aspect of adding a psychiatrist in an adapted shifted outpatient collaborative model, the vast majority of tenant support continued to be delivered by YWCA and Jean Tweed staff. Staff indicated that they benefited from formal and informal support from the psychiatrist. Staff in permanent supportive housing settings face multiple challenges, including high levels of burn-out, inadequate time for training, and high rates of turnover [7]. Our evaluation was not designed to determine whether the collaborative supports were effective at reducing burn-out and turnover; future work could consider exploring this question.

Several of the factors that facilitated implementation reflect those identified in prior research. For example, formal partnerships between agencies, interagency training, case conferencing, and shared care plans were found to be helpful in Australian initiatives to develop integrated housing and mental health services for individuals with psychiatric disabilities [29, 30]. Similarly, the barriers we identified are common to collaborative initiatives in housing settings with multiple agencies, e.g. different workflows across teams when [30], and the ways in which we were able to overcome them could be helpful for other programs aiming to implement similar programs. The interpersonal elements that were key to facilitating implementation (i.e. having a psychiatrist who was a good fit for the setting, allowing time to build trust) have not been previously described in detail in such settings to our knowledge. Trust-building was likely aided by the history of successful collaboration with primary care from the academic hospital, on which psychiatric support could be scaffolded.

Several important contextual elements should be noted in interpreting results. Women’s College Hospital, the YWCA, and the Jean Tweed Centre all have a specific focus on providing women-centered services, and the Elm Centre’s mandate is housing for women and gender diverse people and their children. Similar to in other women-centered housing interventions [31], Elm Centre tenants have high rates of past trauma, and trauma-informed care facilitated implementation. Second, the Elm Centre has a core focus on providing housing for Indigenous woman and gender-diverse people. Indigenous conceptualizations of mental health (which themselves are diverse), often center physical, emotional, mental, and spiritual interconnectedness, while psychiatry is rooted in a Euro-centric Western paradigm, which often favours neurobiological conceptualizations [22, 32]; this disconnect was raised in the focus groups. The psychiatric care in this initiative was intended to complement, rather than replace, existing Indigenous-centered services. Further work is needed to fully understand the needs and perspectives of Indigenous tenants in supportive housing settings to guide future interventions. Finally, this initiative involved three separate agencies, each with its own mandates, leadership, and processes, which added complexity and contributed to implementation barriers.

There were several limitations to this evaluation. As described, there were several changes to the initiative during the evaluation period; while these changes enhanced the quality and relevance of the initiative, it does make evaluation more challenging. We had an ambitious number of data collection forms; and for some of these, we had little to no uptake from participants. With a low response from tenants, the tenant voice was not as prominent as we wanted. We also decided to defer engagement with external primary care providers; this is an important area for future focus and exploration. We relied heavily on de-identified data, and do not have detailed demographic data for tenants/participants; this would be important to include in any future effectiveness studies. The Elm Centre has a specific focus on Indigenous housing, and future work to understand participation and experiences of Indigenous tenants is warranted. The focus of the evaluation was on the addition of the collaborative psychiatrist care, and we did not collect data on the other types of services each tenant was receiving; this would be important to consider in future studies. In the analysis of barriers and facilitators, themes were emergent; as an established implementation science framework was not used to systematically identify barriers and facilitators, some may be underreported. Finally, while this was a hospital-community partnership and efforts were made to engage community partners at all stage of evaluation process, the evaluation was led by individuals affiliated with the hospital which may have shaped the context of the evaluation.

## Conclusion

People living in supportive housing settings have complex mental health needs, and existing services do not always meet these needs. Here, we describe a collaborative approach in which team-based mental health support, tenant engagement, and support for staff was delivered via the addition of a one-day-per-week psychiatrist to existing housing and case management supports. We identified several facilitators and barriers to implementation, which can be used to inform mental health program development and implementation in other permanent housing settings. Future work is needed to evaluate the effectiveness of this intervention with respect to tenant, staff, and housing complex outcomes; if effective, work is needed to evaluate its scalability and applicability to other supportive housing contexts.

## Supplementary Information

**Additional file 1: Figure S1.** Study measures for each aim, and adaptations made. **Figure S2.** Logic model. **Table S1.** Staff 9-month survey. **Table S2.** Qualitative feedback related to shared lenses.

## Data Availability

The data supporting the conclusions of this article are not publicly available. Data are available from the corresponding author upon reasonable request.
