# Supplementary material for: Delivering collaborative mental health care within supportive housing: implementation evaluation of a community-hospital partnership
Source: BMC Psychiatry. 2022 Jan 13;22:36. doi: 10.1186/s12888-021-03668-3 (PMC8756167; doi:10.1186/s12888-021-03668-3)
Supplement: Supplementary file 1 — Additional file 1: Figure S1. Study measures for each aim, and adaptations made. Figure S2. Logic model. Table S1. Staff 9-month survey. Table S2. Qualitative feedback related to shared lenses. [file 12888_2021_3668_MOESM1_ESM.docx]

**Supplemental Materials**

Figure S1. Study measures for each aim, and adaptations made. Domains are according to Saunders et al.[32]

^1^Likert ratings on a 1-5 scale

^2^Program documents include (a) minutes from stakeholder meetings, (b) weekly and monthly psychiatrist documentation, (c) published materials related to the three partner organizations (e.g. websites)

^3^PCP – After starting the initiative, it was decided to prioritize collaboration with on-site staff, and PCP engagement was challenging due to PCPs being offsite and a gap in which PCP coordinator role was not filled.

^4^Tenants did not complete initial surveys so was unable to measure this. Second survey for rostered tenants was replaced with a survey for all tenants to learn how to improve patient-centeredness.

^5^Psychiatrist documentation was for less than one year because APQIP approval for the documentation form was obtained in August 2019, and regular initiative activities were suspended in March 2020 due to COVID-1

Figure S2. Logic model^1^

^1^In this evaluation, the focus was on the process of implementing program activities, not on measuring effectiveness outcomes.

**Table S1. Staff 9-month survey (n=10)^a^**

| **Questions** | **Median^b^ (Interquartile range)** |
| --- | --- |
| I feel satisfied with the Elm-Women's care team. | 3 (3-4) |
| Working in Elm-Women's is collaborative. | 3.5 (3-4) |
| I feel engaged in the Elm-Women's care team. | 4.5 (4-5) |
| The care we provide in Elm-Women's is patient-centered. | 4 (4-4) |
| The care we provide in Elm-Women's is trauma-informed. | 4 (3.25-4) |
| The care we provide in Elm-Women's is culturally appropriate. | 4 (3.35-4.75) |
| The care we provide in Elm-Women's takes a harm reduction approach. | 3.5 (3-4) |

^a^A survey was also conducted at 3 months, however the number of respondents was too small for it to be reliably interpreted.

^b^Responses: 1=Strongly disagree; 2=Disagree; 3=Neutral; 4=Agree; 5=Strongly Agree

**Table S2. Qualitative feedback related to shared lenses**

| **Lens** | **Selected Quotes** |
| --- | --- |
| **Trauma-informed care** | - “The client was so comfortable upon meeting her, and she was so just gentle with the client that the client ended up opening up quite quickly about some past trauma. It was really, really nice, and I just felt that client felt, I don’t even know how to describe it, it was really good, and that client hasn’t had doctors or psychiatrists who she has felt that connected with and able to do that.” (Staff 6-month focus group) - “I think having Dr. X and Dr. X around has really helped build our capacity for trauma-informed care, having that understanding, having their clinical perspective around things, and bringing that lens into our focus… really collaborating around that piece has helped us really build skills under the trauma-informed umbrella in our work”. (Staff final focus group) - “…we’re really big on being trauma informed, and that’s something that has always been just a thing. And I guess I feel that Dr. X certainly brings that as well. And if anything, has just added to what we already were trying to do or practice here with being trauma informed.” (Staff final focus group) |
| **Culturally safe care** | - “I think there’s definitely still room to grow in some of our cultural aspects. I was seeing that start to play out a little bit more with the group, given that a large number of tenants that come to us are from the Indigenous community, and so have a very different understanding of mental health, and healing, and growth in that sense.” (Staff final focus group) - “I think when you’re talking about how to work across cultures, it really boils down to respect. And I definitely got that sense from [the psychiatrist] and how Women’s College Hospital wants to work with us and the community that we serve.” (Staff final focus group) - “And I think Dr. X herself acknowledges that and has acknowledged that, that [psychiatry]’s a very Westernized, very specific way of viewing mental health. And that she never was like, this is the only way to talk about mental health.” (Staff final focus group) |
| **Harm Reduction** | - **“**We are a harm-reduction program, and in some ways, I find we excel at that harm-reduction aspect, and other ways not so much. But having Dr. X around and available has definitely contributed to our ability to work more from a harm-reduction perspective.” (Staff final focus group) - “Dr. X, especially I think her background of trauma also kind of blends in with the harm reduction background in terms of understanding why people do certain behaviours, where that is rooted from and coming from a place of non-judgement, knowing that it serves a purpose for each individual or if they’re getting a need met in some way... [The tenants] reflected feeling that it was a very safe person for them to talk to because they didn’t feel like there was any judgement. It was more just like, this is information. You get to make your choice.” (Staff 6-month focus group) |
